# Supplementary material for: Specific ion effects at graphitic interfaces
Source: Nat Commun. 2019 Oct 24;10:4858. doi: 10.1038/s41467-019-12854-7 (PMC6813325; doi:10.1038/s41467-019-12854-7)
Supplement: Supplementary file 1 — Supplementary Information [file 41467_2019_12854_MOESM1_ESM.pdf]

Specific ion effects at graphitic interfaces

Zhan *et al.*

Supplementary Table 1: Capacitance measured for HCAMs electrode in 1 M aqueous solutions at a scan rate of 0.5 mVs^{-1}. Ionic radius, hydration radius of Li^+, Na^+, K^+ and Cs^+ and their calculated adsorption energy on a graphene basal plane with different charges, including $0e$, $-0.5e$, $-1.0e$ and $-1.5e$, are also included. For the calculations of cations at the interface with graphene, we model the electrode by a 96-atom orthorhombic cell, as discussed in the main text. For the binding energy, negative values indicate a favorable interaction between the cations and graphene.

Ions	Ionic radius[1] (Å)	Hydrated radius[1] (Å)	Adsorption energy (eV)	Capacitance (Fg^{-1})
Li^+	0.60	3.82	+0.517/+0.347/+0.276/+0.241	82.89
Na^+	0.95	3.58	+0.198/+0.119/+0.069/-0.020	84.05
K^+	1.33	3.31	+0.074/+0.004/-0.068/-0.106	86.59
Cs^+	1.69	3.29	-0.004/-0.078/-0.183/-0.263	93.04

Supplementary Table 2: Current (mA) and capacitance (Fg^{-1}) dependence of HCAMs electrode on potential sweep rates using two-electrodes set up for 50 mM LiCl, NaCl, KCl and CsCl electrolytes.

Salts 50 mM	Current 0.5 mVs^{-1}	Capacitance 0.5 mVs^{-1}	Current 1.0 mVs^{-1}	Capacitance 1.0 mVs^{-1}	Current 1.5 mVs^{-1}	Capacitance 1.5 mVs^{-1}
LiCl	2.82	75.58±0.082	5.20	69.79±0.056	7.27	65.04±0.01
NaCl	2.97	79.70±0.081	5.50	73.78±0.051	7.73	69.15±0.01
KCl	2.97	79.78±0.060	5.58	74.78±0.007	7.88	70.53±0.04
CsCl	3.08	82.57±0.061	5.79	74.95±0.043	8.23	73.68±0.02

Supplementary Table 3: Current (mA) and capacitance (Fg^{-1}) dependence of HCAMs electrode on potential sweep rates using two-electrodes set up for 1 M LiCl, NaCl, KCl and CsCl electrolytes.

Salts<br>50 mM	Current<br>0.5 mVs^{-1}	Capacitance<br>0.5 mVs^{-1}	Current<br>1.0 mVs^{-1}	Capacitance<br>1.0 mVs^{-1}
LiCl	3.36	82.89±0.067	6.70	82.55±0.006
NaCl	3.41	83.05±0.044	6.73	82.90±0.021
KCl	3.51	86.59±0.053	6.98	86.05±0.023
CsCl	3.77	93.04±0.070	7.53	92.74±0.011

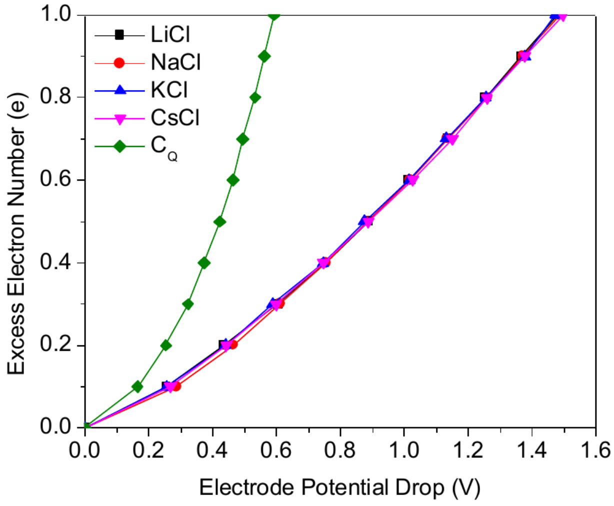

Supplementary Fig. 1: Comparison of the charging behaviors of the graphene electrode in different aqueous solutions within a voltage window determined by an excess charge of [0:1]× e. The total charge-voltage potential (CV) dependencies computed for LiCl, NaCl, KCl, and CsCl are represented by black, red, blue and magenta lines, respectively. The CV associated to the quantum capacitance contribution is also shown by green line. The theoretical capacitances computed at the $-1e$ charge state is 4.36, 4.22, 4.09 and 4.06 μFcm^{-2} for LiCl, NaCl, KCl, and CsCl solution, respectively. The results are obtained using the computational approach presented in our previous study.[2]

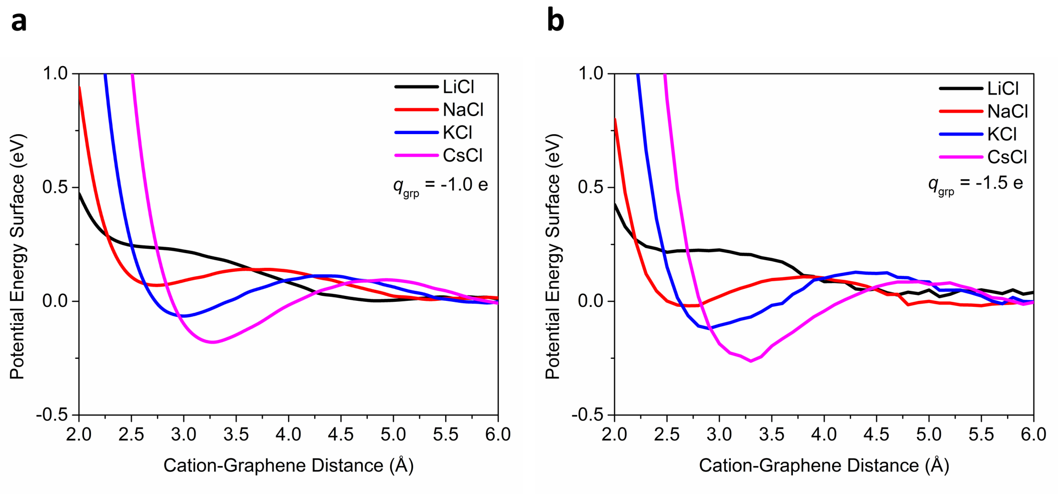

Supplementary Fig. 2: Calculated potential energy surface (PES) of the adsorption of alkali metal ions on a charged graphene electrode. The total excess charge of the electrode is $-1e$ (in **a**) and $-1.5e$ (in **b**).

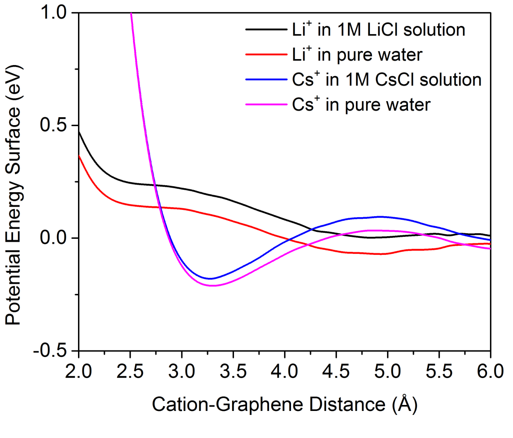

Supplementary Fig. 3: Calculated potential of energy surface (PES) for explicit Li^+ and Cs^+ on a charged graphene with a charge density of $-0.0052e$ per carbon atom. The calculations were carried out for 1 M aqueous solutions and pure liquid water.

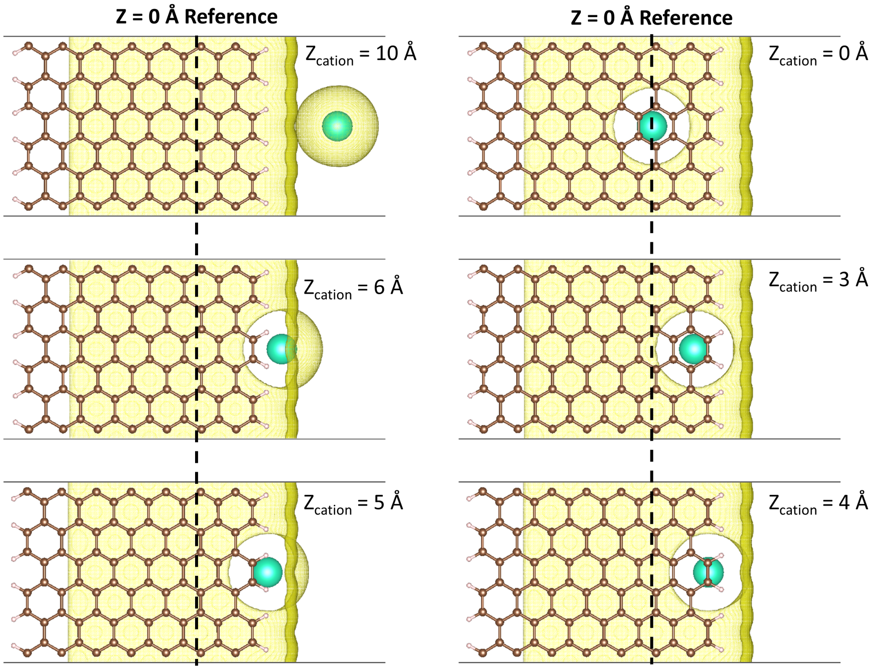

Supplementary Fig. 4: Iso-surfaces of oxygen density representing solvation structure of Cs^+ during intercalation, where z is the distance between ion and the surface hydrogen. In particular, configuration at $z = 10$ Å represents solvated Cs^+ in bulk electrolyte; configurations at 6.0, 5.0, and 4.0 Å from the surface represent partially desolvated Cs^+ at the interface, whereas those at 3.0 Å and 0.0 Å correspond to fully desolvated Cs^+ inside the pore.

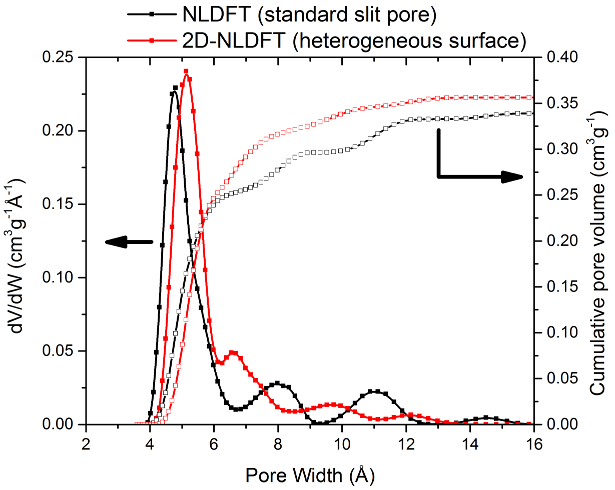

Supplementary Fig. 5: Pore size characteristics of the hierarchical carbon aerogel monoliths (HCAMs), including volume derivative with respect to the pore width, and cumulative pore volume. To measure the micro-porosity, we carried out N_2 absorption at 77 K with a Micromeritics ASAP 2020. In addition, in order to adequately resolve the micropore structure, the experiment was conducted over a 100 h time period to allow the N_2 time to diffuse into the narrow porous regions. The final pore size distribution (PSD) was obtained using both the NLDFT Standard Slit model and the 2D-NLDFT Heterogeneous Surface model[3] processed in the SAIEUS software package.[4,5] The resulting PSD plots show substantial similarities between the two models, with the key feature being the majority of pore volume located at ∼5-6 Å.

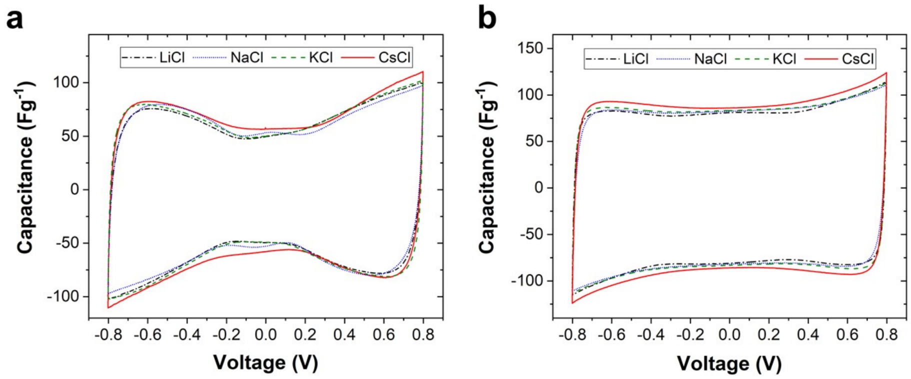

Supplementary Fig. 6: *CV* measurements of HCAMs electrodes in (**a**) 50 mM and (**b**) 1.0 M electrolytes. The scan rate in all measurements is 0.5 mVs^{-1}. The calculated capacitance is summarized in Supplementary Tables 2 & 3.

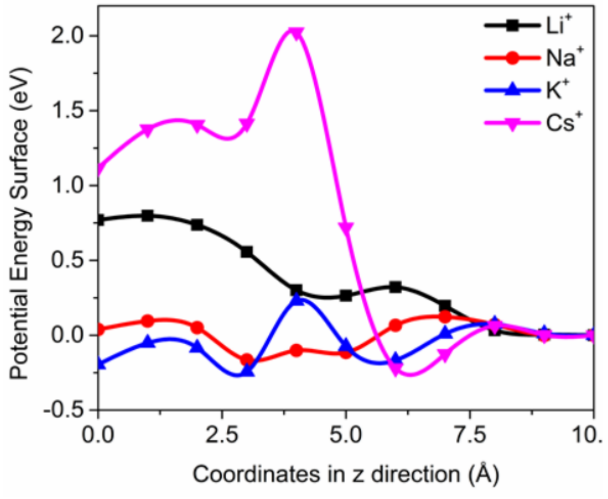

Supplementary Fig. 7: Calculated potential of energy surface (PES) for alkali metal cations intercalation into a graphene slit-pore with a pore size of 5 Å.

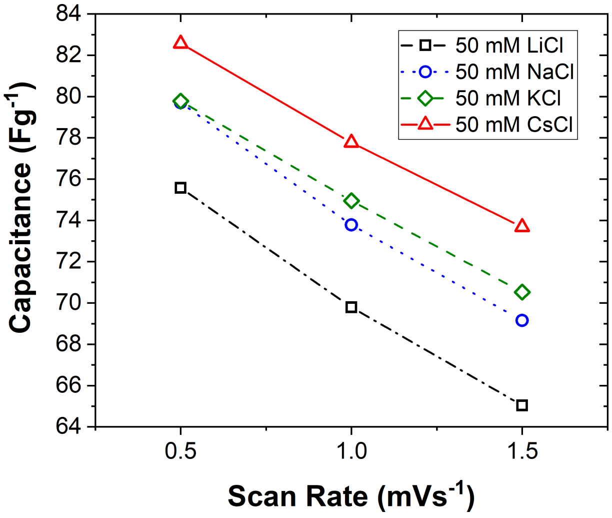

Supplementary Fig. 8: Capacitance dependence on potential sweep rates using two-electrodes set up and 50 mM LiCl, NaCl, KCl and CsCl electrolytes.

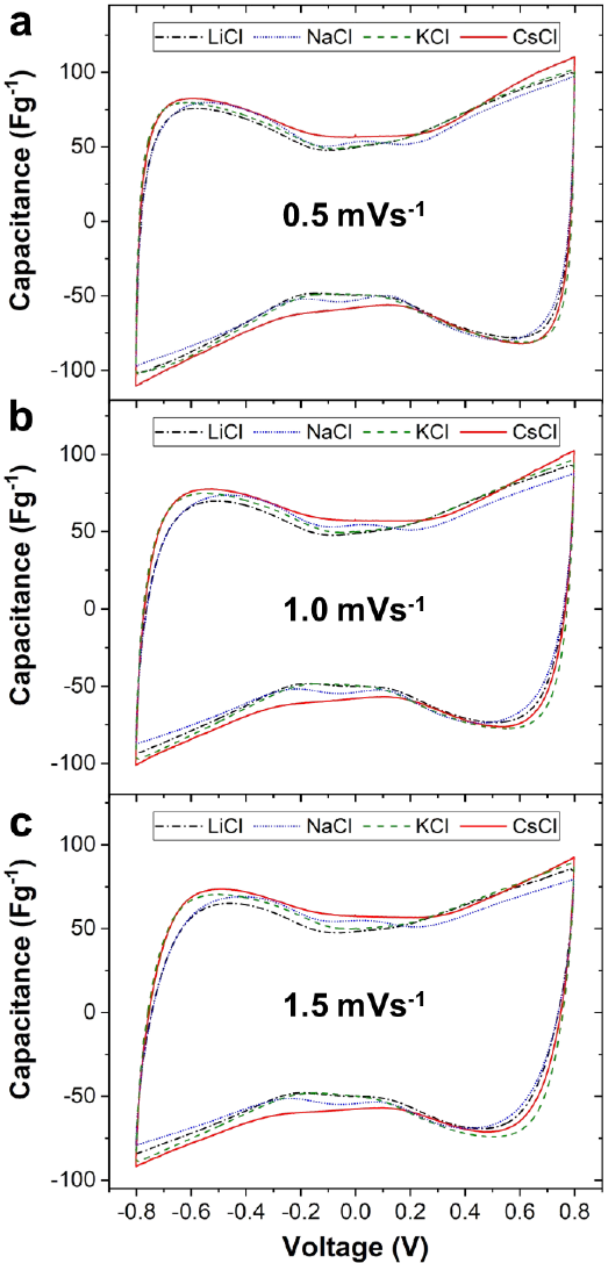

Supplementary Fig. 9: Cyclic voltammogram of two carbon aerogel electrodes using 50 mM LiCl, NaCl, KCl and CsCl electrolytes at (a) scan rate 0.5 mVs^{-1}; (b) scan rate 1.0 mVs^{-1}; and (c) scan rate 1.5 mVs^{-1}. The calculated capacitance is summarized in Supplementary Tables 2 & 3.

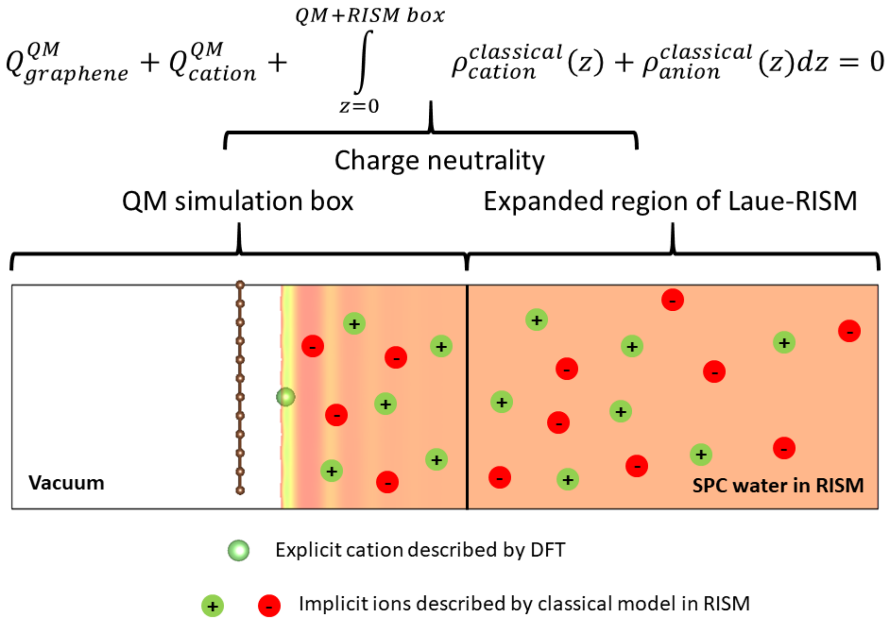

Supplementary Fig. 10: A schematic description of our hybrid quantum-continuum approach.[6] The explicit cations and carbon electrodes were described at the DFT level of theory, whereas the electrolyte was represented by an implicit solvent model consisting of water molecules and 1 M of salt. The interaction between the explicit "graphene-cation" system and the implicit solution is described through the Lennard-Jones (LJ) and electrostatic potentials. In particular, the electrostatic potential acting on the implicit solution species is computed directly from first-principles, whereas the LJ parameters of universal force fields were employed for the carbon atoms. Atomic charges and LJ potentials of the classical solvent and ions are described through the OPLS all-atom force fields.[7,8] Our calculations of cations on charged electrodes were carried out by fixing the net charge of the explicit quantum mechanical region, which is balanced by the classical ions in the RISM solvation, and therefore the whole system is neutral.

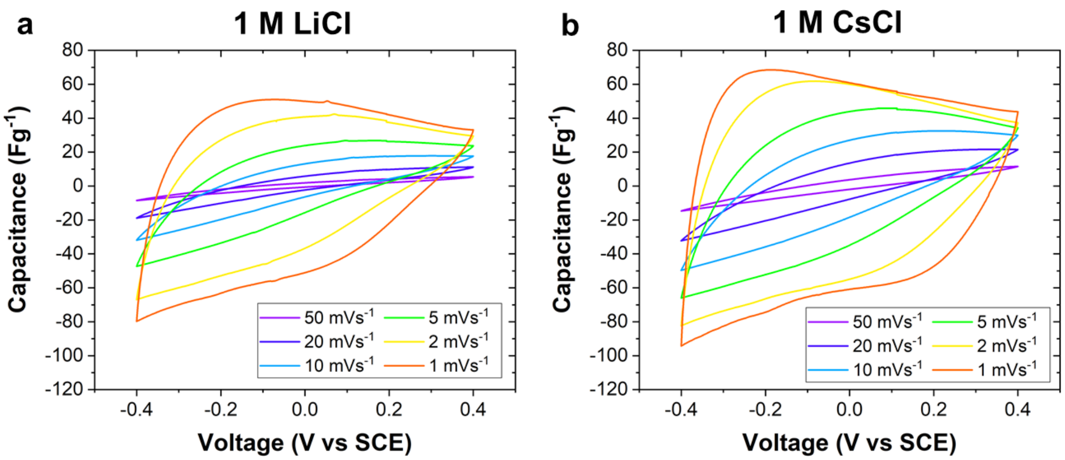

Supplementary Fig. 11: Cyclic voltammogram of three-electrodes set up at different scan rates, electrolyte (a) 1 M LiCl (b) 1 M CsCl.

Supplementary Methods

Due to the high resistant in thick monolithic activated HCAM electrode systems,[9–11] measuring cyclic voltammetry at higher scan rates provide less accurate capacitance information. The *CV* of 1 M LiCl and CsCl at different scan rates was measured using a three-electrode setup, particularly at scan rates below 10 mVs^{-1} where more accurate capacitance values can be determined, and we observed similar trend compared to the two-electrodes experiments.

We also note that the calculated capacitance with the three-electrodes is lower than the one obtained with the two-electrode set up, this can be explained by the higher resistance observed in the three-electrode measurements, due to poor contact between our thick monolithic electrode and the current collector, as well as the differences in the electrode geometry of both methods. Most importantly, the calculated capacitance with the two electrode measurements accounts for both the cation and the anion, while the calculated capacitance with the three-electrode measurements accounts for the cation or the anions depending on the potential polarity. The values plotted on Supplementary Fig. 13 is only for the cation ions.

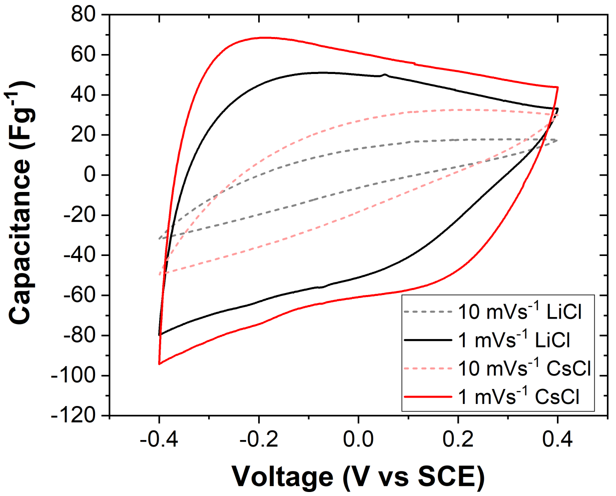

Supplementary Fig. 12: Cyclic voltammogram of three-electrodes set up at 10 and 1 mVs^{-1} using 1 M LiCl and CsCl electrolytes. Carbon aerogel working electrode, platinum wire counter electrode, calomel reference electrode.

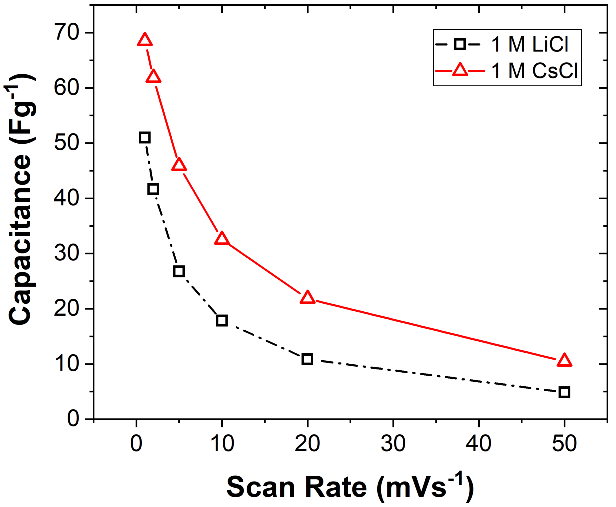

Supplementary Fig. 13: Capacitance dependence on potential sweep rates using three-electrodes set up and 1 M LiCl and CsCl electrolytes.

Supplementary references

(1) Nightingale Jr, E. Phenomenological theory of ion solvation. Effective radii of hydrated ions. *J. Phys. Chem.* **1959**, *63*, 1381–1387.

(2) Zhan, C.; Pham, T. A.; Cerón, M. R.; Campbell, P. G.; Vedharathinam, V.; Otani, M.; Jiang, D.-e.; Biener, J.; Wood, B. C.; Biener, M. Origins and Implications of Interfacial Capacitance Enhancements in C60-Modified Graphene Supercapacitors. *ACS Appl. Mater. Interfaces* **2018**, *10*, 36860–36865.

(3) Jagiello, J.; Olivier, J. P. 2D-NLDFT adsorption models for carbon slit-shaped pores with surface energetical heterogeneity and geometrical corrugation. *Carbon* **2013**, *55*, 70–80.

(4) Jagiello, J. Stable numerical solution of the adsorption integral equation using splines. *Langmuir* **1994**, *10*, 2778–2785.

(5) SAIEUS software downloaded from http://www.nldft.com.

(6) Nishihara, S.; Otani, M. Hybrid solvation models for bulk, interface, and membrane: Reference interaction site methods coupled with density functional theory. *Phys. Rev. B* **2017**, *96*, 115429.

(7) Jorgensen, W. L.; Tirado-Rives, J. The OPLS [optimized potentials for liquid simulations] potential functions for proteins, energy minimizations for crystals of cyclic peptides and crambin. *J. Am. Chem. Soc* **1988**, *110*, 1657–1666.

(8) Jorgensen, W. L.; Maxwell, D. S.; Tirado-Rives, J. Development and testing of the OPLS all-atom force field on conformational energetics and properties of organic liquids. *J. Am. Chem. Soc* **1996**, *118*, 11225–11236.

(9) Pell, W.; Conway, B.; Marincic, N. Analysis of non-uniform charge/discharge and rate

effects in porous carbon capacitors containing sub-optimal electrolyte concentrations. *J. Electroanal. Chem.* **2000**, *491*, 9–21.

(10) Pell, W. G.; Conway, B. E. Analysis of power limitations at porous supercapacitor electrodes under cyclic voltammetry modulation and dc charge. *J. Power Sources* **2001**, *96*, 57–67.

(11) Niu, J.; Pell, W. G.; Conway, B. E. Requirements for performance characterization of C double-layer supercapacitors: Applications to a high specific-area C-cloth material. *J. Power Sources* **2006**, *156*, 725–740.
